# How Non-Uniform Stiffness Affects the Propulsion Performance of a Biomimetic Robotic Fish

**DOI:** 10.3390/biomimetics7040187

**Published:** 2022-11-03

**Authors:** Changzhen Zheng, Jiang Ding, Bingbing Dong, Guoyun Lian, Kai He, Fengran Xie

**Affiliations:** 1 School of Artificial Intelligence, Shenzhen Polytechnic, Shenzhen 518055, China; 2 College of Mechanical Engineering, Guangxi University, Nanning 530004, China; 3 Shenzhen Institute of Advanced Technology, Chinese Academy of Sciences, Shenzhen 518055, China; 4 College of Mechanical Automation, Wuhan University of Science and Technology, Wuhan 430000, China

**Keywords:** biomimetic robotic fish, non-uniform stiffness, propulsion performance

## Abstract

Live fish in nature exhibit various stiffness characteristics. The anguilliform swimmer, like eels, has a relatively flexible body, while the thunniform swimmer, like the swordfishes, has a much stiffer body. Correspondingly, in the design of biomimetic robotic fish, how to balance the non-uniform stiffness to achieve better propulsion performance is an essential question needed to be answered. In this paper, we conduct an experimental study on this question. First, a customized experimental platform is built, which eases the adjustment of the non-uniform stiffness ratio, the stiffness of the flexible part, the flapping frequency, and the flapping amplitude. Second, extensive experiments are carried out, finding that to maximize the propulsion performance of the biomimetic robotic fish, the non-uniform stiffness ratio is required to adapt to different locomotor parameters. Specifically, the non-uniform stiffness ratio needs to be reduced when the robotic fish works at low frequency, and it needs to be increased when the robotic fish works at high frequency. Finally, detailed discussions are given to further analyze the experimental results. Overall, this study can shed light on the design of a non-uniform biomimetic robotic fish, which helps to increase its propulsion performance.

## 1. Introduction

Through millions of years of evolution, fish have possessed remarkable swimming performance. They can cross miles of ocean in search of habitat or maneuver quickly to prey. The high efficiency, low energy consumption, and high mobility of their movements are mainly due to their streamlined shapes, strong muscle, and ability to modulate the stiffness of the body [1,2,3]. In nature, the body of a live fish is made of collagen, bones, ligaments, and muscle fibers, which form a unique structure with certain rigidity and flexibility at the same time. This biological feature provides an ability to adjust their body stiffness on their own. In contrast, most of the biomimetic robotic fishes at present are rigid, which may be one of the reasons why their swimming performance is far inferior to that of live fish. In recent years, to solve the problem of low propulsion and driving efficiency of biomimetic robotic fish, Lauder et al. [4], Shelton et al. [5], and Feilich et al. [6] explored the effect of uniform stiffness on the propulsion performance of robotic fish. They found that increased stiffness will cause an increase in thrust coefficient, propulsion efficiency, and swimming speed under certain conditions. From the bionic perspective of real fish with decreasing stiffness along the head to the caudal fin, the literature [7,8,9,10,11,12,13] proposed to rely on non-uniform stiffness to enhance the driving force. Moore et al. [7], Yeh et al. [8], and Zhu et al. [9] demonstrate that uniform stiffness produces inferior propulsive effects to non-uniform stiffness. Lucas [10] et al. conducted a series of comparative experimental studies of propulsive force, efficiency, and energy consumption using four non-uniform models. However, it only focused on the length ratio of the rigid foil to the soft foil. The stiffness (or thickness) of the soft part was not investigated. The literature [11,12,13] has experimentally explored the stiffness distribution based on numerical optimization analysis, including uniform distribution, declining distribution, and growing distribution. It has been demonstrated that the declining stiffness distribution, the rigid anterior border, and the flexible posterior edge, maximize the propulsive force. Zhong [14] et al. have equipped the “artificial muscles” for robotic fish in hope that they can adjust their stiffness autonomously like their counterpart in nature. However, among these studies, the questions of how to design the length ratio of the rigid part to the soft part and how to choose the optimal stiffness to drive the robotic fish, have not been fully answered.To find the optimal stiffness, it is almost impossible to obtain the final experimental results directly through real fish experiments. The main reason is that the real fish cannot accurately measure the propulsion force and torque generated by the fin during the experiments. There is also no guarantee that the initial motion state is consistent in each experiment, which undoubtedly affects the final experimental results. In addition, the propulsion force of the biomimetic robotic fish is affected by a variety of factors, such as its bionic shape, material, stiffness of fins, etc. Single-factor kinetic and kinematic studies using three-dimensional fish bodies would make the experimental and mechanical analysis process extremely complex [15,16] and may distort the experimental results by the non-research factors. Therefore, numerous researchers have proposed an idea for single-factor studies using a simple mechanical model [17,18,19,20,21,22,23,24,25,26,27,28], which would greatly simplify the experimental process and make the experimental results more realistic and valid.

In this paper, we perform an experimental study on how non-uniform stiffness affects the propulsion performance of a biomimetic robotic fish. A customized experimental platform is built and extensive experiments are carried out. The contributions of this paper are twofold. On one hand, both the non-uniform stiffness ratio and the stiffness of the flexible part are investigated in the nonuniform flapping model for the first time. On the other hand, it is interesting to find that to maximize the propulsion performance of the biomimetic robotic fish, the non-uniform stiffness ratio needs to be reduced when the robotic fish works at low frequency, and it needs to be increased when the robotic fish works at high frequency.

The rest of this paper is organized as follows. Section 2 describes the materials and methods, i.e., the structural design. Section 3 presents the experimental results and related analyses. Section 4 provides a further discussion. Finally, Section 5 gives a systematic summary of this article and an outlook on future research directions.

## 2. Materials and Methods

**Figure 1:**
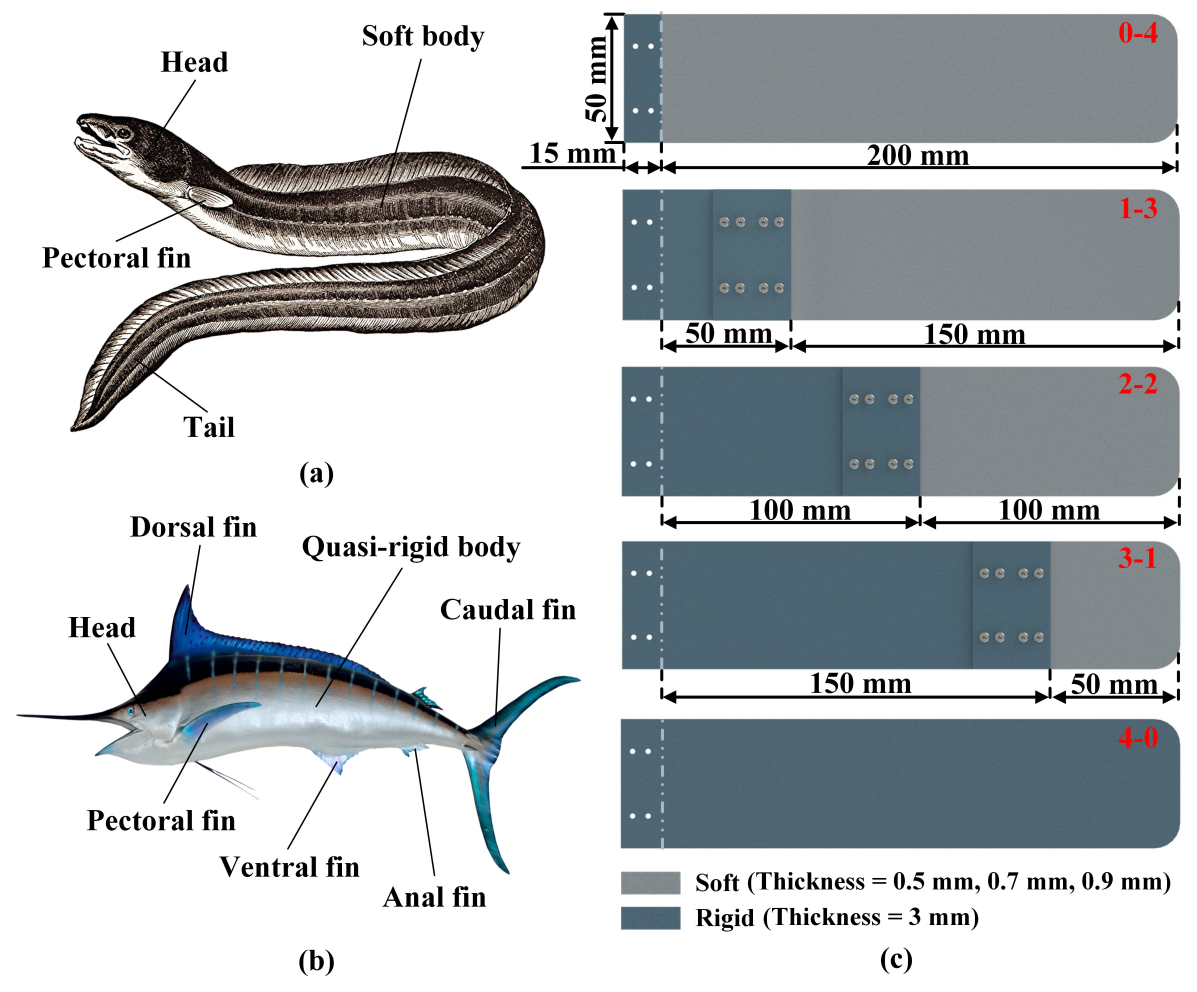
Biomimetic structural and mechanism models: (**a**) The eel with a flexible body. (**b**) The swordfish with a quasi-rigid body, whose swimming speed can reach 130 km/h. (**c**) The mechanism models: There are five kinds of non-uniform stiffness models. The light gray color represents the flexible material and the dark gray color represents the rigid material, and the thickness of the flexible material is 0.5 mm, 0.7 mm, and 0.9 mm, and the thickness of the rigid material is 3 mm.

It is known that the eel has a soft body, as shown in Figure 1a, which makes it feature a certain degree of motor flexibility, but relatively poor maneuverability. Compared with the eel, the body of the swordfish tends to be rigid, as shown in Figure 1b, with a swimming speed of 130 km/h [29], but its locomotor flexibility is much less than that of the eel [30,31,32]. In addition, according to the distribution characteristics of the stiffness of the real fish, and inspired by the above biological constitution of eels and swordfishes, we designed a mechanism model with a rigid anterior border and flexible rear border, namely a non-uniform stiffness model.

The mechanism model is shown in Figure 1c. The light gray part represents flexible plates (thickness = 0.5 mm, 0.7 mm, 0.9 mm) made of polypropylene. Rigid plates (thickness = 3 mm) made of aluminum alloy are indicated in dark gray. The length of the entire foil is 200 mm and the width is 50 mm. In addition, an articulation plate of size 15 mm is reserved at the leading edge of the foil for the rigid connection between the foil and the rudder. The structure is fastened with 2 mm diameter bolts. There are five kinds of the mechanism model, corresponding to different non-uniform stiffness ratios of foils. The designation is given according to the rule of rigid-flexible percentage. For example, the first flapping foil (pure flexible foil) in Figure 1c is named 0-4, where 0 represents the ratio of rigid material of the whole foil and 4 is the ratio of flexible material of the foil. In other words, the length of the rigid part of the low stiffness (0-4) is 0 mm, and the length of the flexible part is 200 mm. The remaining flapping foils are labeled 1-3, 2-2, 3-1, and 4-0, respectively. In addition, the rigid plate and the flexible plate are linked together by two rigid sheets, which is similar to a sandwich structure. To prevent the foil from loosening during the experiment and thus affecting the experimental results, the coupling plate was fastened by 8 bolts of 2 mm diameter. The Young’s modulus of the soft foil is 1356 Mpa. The stiffness of the flexible portion of models with thicknesses of 0.5 mm, 0.7 mm, and 0.9 mm are $EI_{0.5}$ = 7.06 × $10^{-4}$ Nm^2^, $EI_{0.7}$ = 1.94 × $10^{-3}$ Nm^2^, and $EI_{0.9}$ = 4.12 × $10^{-3}$ Nm^2^, respectively.

Please note that the effect of the mass distribution of the beam on the fluid-structure interactions can be neglected for two main reasons. On one hand, the servo motor in this paper works in position control mode. It can eliminate the influence of some disturbing factors, such as beam mass distribution and vibration. For example, when the foil weighs down or weighs up, the servo motor can decrease or increase its torque corresponding, which overcomes this factor and ensures that the foil can bend to the target position. On the other hand, the combination of rigid foil and servo motor is considered as a rigid base, which is capable to output rotational motion. Therefore, if we want to know the effect of the fluid-structure interactions on the propulsion performance, we need to focus more on the flexible foil part, while the mass distribution of the flexible foil is uniform.

## 3. Experiments

**Figure 2:**
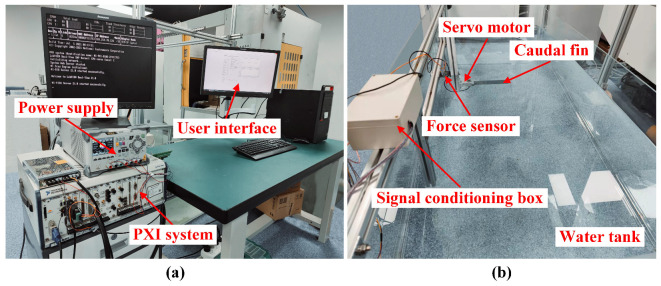
The experimental setup: (**a**) The motion control unit of the platform. (**b**) The foil flapping in a water tank of the size of 2000(L) × 1000(W) × 600(H) mm.

The experimental platform is shown in Figure 2, where Figure 2a displays the motion control unit, which mainly includes a computer where the user interface runs, a power supply, and a PXI system providing control signals for the servo motor. Figure 2b shows an image of the foil flapping in a water tank of the size of 2000(L) × 1000(W) × 600(H) mm. In addition, we applied the sinusoidal equation to modulate the motion parameter of the servo motor. The model is as shown below:

(1) $$\theta \left(t\right)=\theta_{a}\sin\left(2\pi ft+\theta_{i}\right)$$

where θ(t) represents the pitch angle, $\theta_{a}$ is the pitch amplitude, *f* represents the pitch frequency, and $\theta_{i}$ is the initial phase.

The experiments are divided into three parts based on different flapping frequencies. As a whole, the movement frequency of the foil is set at 0.5 Hz in the first group, the second and third groups are set at 1.0 Hz and 1.5 Hz, respectively. The flapping amplitude of the foil in each group of the experiments is set to the range of 20° to 50° with an interval of 10°. In addition, the foil is assembled by matching five kinds of non-uniform stiffness ratios and three different thicknesses of flexible plates in the experiment. There are rigid plates with a thickness of 3 mm and the thicknesses of flexible plates are 0.5 mm, 0.7 mm, and 0.9 mm respectively. To minimize the experimental error, each experiment is repeated three times, and it is conducted when the water surface is close to stationary.

### 3.1. Experiment I (Frequency = 0.5 Hz)

**Figure 3:**
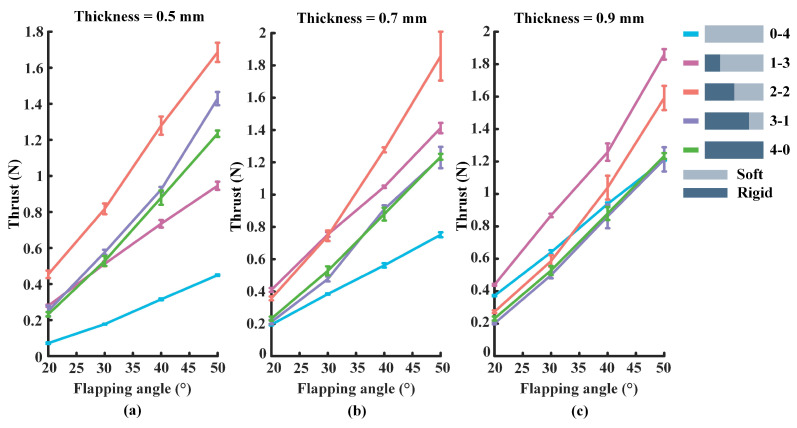
The thrust produced by the foil at the flapping frequency of 0.5 Hz: (**a**) The thickness of the flexible plate of the foil is set to 0.5 mm. (**b**) The thickness of the flexible plate is set to 0.7 mm. (**c**) The flexible plate’s thickness is 0.9 mm.

Figure 3 shows a comparison of the propulsive force when the flapping frequency of the foil is set to 0.5 Hz. The vertical coordinate represents the propulsive force generated by the foil and the horizontal coordinate represents the flapping angle of the foil, which is set to 20°, 30°, 40°, and 50°, respectively. In general, the propulsion force will vary in the range of 0 to 2 N, and the trust will increase with the increase of the flapping angle of the foil. Moreover, the legend on the right side clearly shows the correspondence between the individual curve and the mechanism model. Specifically, light gray represents flexible plates, dark gray is rigid plates, and different color lines represent different non-uniform stiffness ratios. To reduce the experimental error, each group of experiments is repeated three times, and the value of each data point is the average of the results of the three experiments.

From Figure 3a, there is a diagram of propulsion force when the flexible plate’s thickness is 0.5 mm and the flapping amplitude is set to 20°, 30°, 40°, and 50°, respectively. The 2-2 Foil produces the relatively largest thrust, while the high stiffness (4-0) foil produces smaller values of propulsive force, and the low stiffness (0-4) foil’s force is the smallest. From Figure 3b, it can be found that the propulsive force of the 2-2 foil is relatively the largest, while low stiffness (0-4) foil produces the smallest trust when the thickness of the flexible plate is 0.7 mm. In Figure 3c, the 1-3 foil (stiff anterior one quarter) produces the relatively largest propulsive force value when the thickness of the flexible plate is 0.9 mm. It is worth noting that the low stiffness (0-4) foil’s thrust tends to increase significantly, while the 3-1 (stiff anterior three-quarters) foil produces the smallest propulsive force.

**Figure 4:**
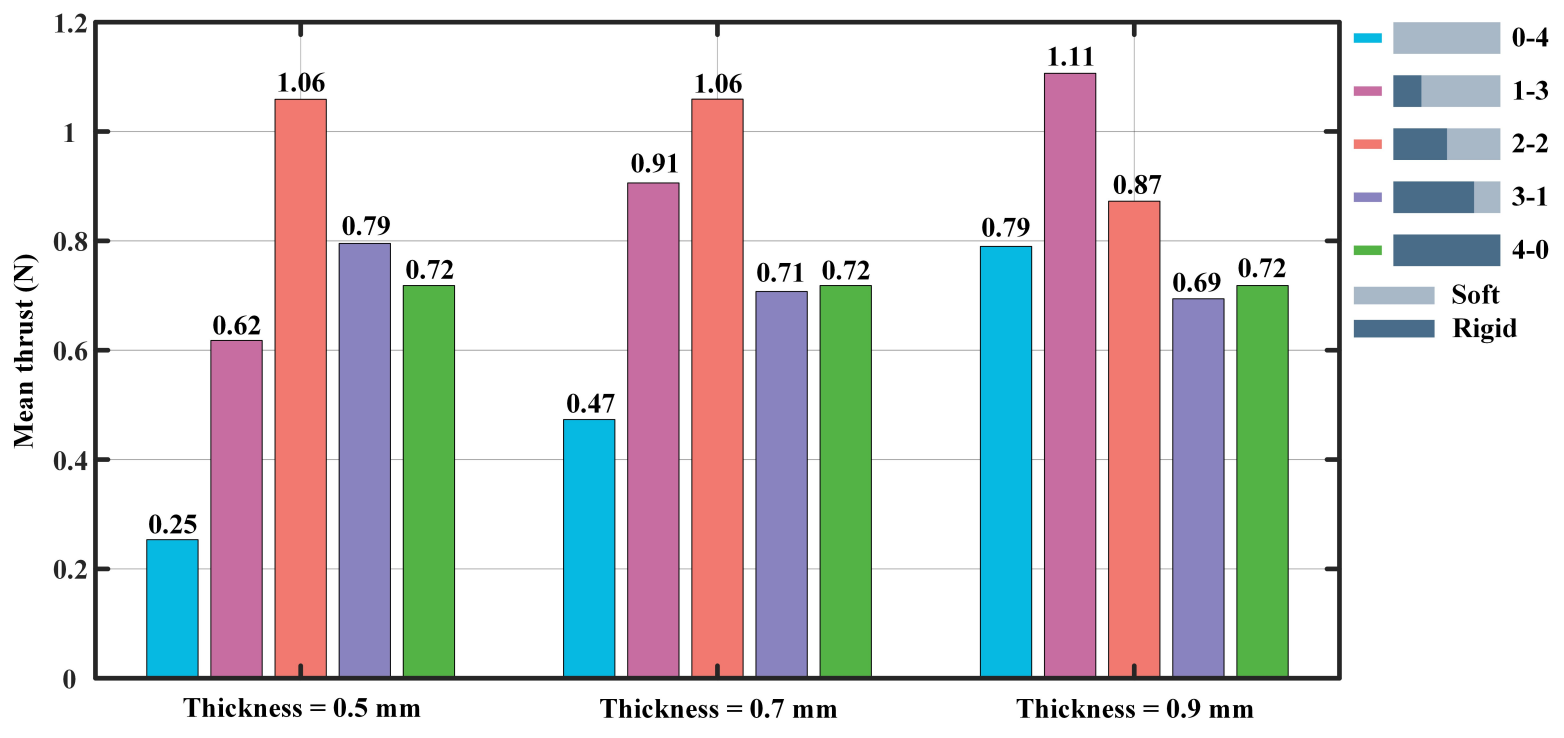
The mean thrust produced by the foil at the flapping frequency of 0.5 Hz, averaged over all flapping angles from 20° to 50°.

Figure 4 shows the mean thrust information for the flapping angle of 20° to 50°. When the thickness of the flexible plate of the foil is 0.5 mm, the 2-2 foil (stiff anterior two quarters) produces the largest propulsive force with a value of 1.06 N, and the low stiffness (0-4) foil’s mean thrust is the minimum of 0.25 N. In addition, when the flexible plate’s thickness is 0.7 mm, the 2-2 foil and 0-4 foil produce the largest and smallest average propulsive forces of 1.06 N and 0.47 N, respectively. Notably, the thickness of the flexible plate is set to 0.9 mm, the 1-3 foil produces the largest average propulsive force of 1.11 N, while the smallest average thrust is 0.69 N, which was generated by the 3-1 foil. As a whole, the propulsive force generated by the 0-4 and 1-3 foils gradually increase as the thickness of the flexible plate increase, while the thrust tends to decrease significantly for the 2-2 and 3-1 foils. In addition, when the flexible plate’s thickness is 0.9 mm, the 1-3 foil produces the largest mean propulsive force of 1.11 N, while the low stiffness (0-4) and high stiffness (4-0) foils provide the mean propulsive forces of 0.79 N and 0.72 N, respectively. It can be seen that the propulsive force produced by the model with rigid-flexible coupled materials is 40% and 54% higher than the counterpart generated by low stiffness (0-4) and high stiffness (4-0) foils, respectively.

### 3.2. Experiment II (Frequency = 1.0 Hz)

**Figure 5:**
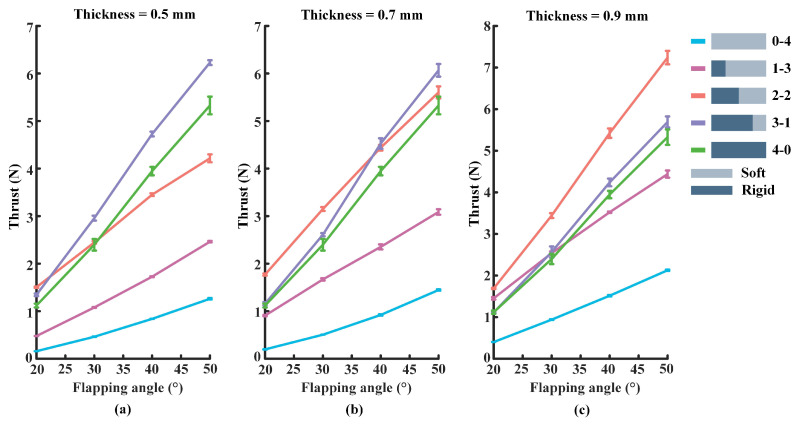
The thrust produced by the foil at the flapping frequency of 1.0 Hz: (**a**) The thickness of the flexible plate of the foil is set to 0.5 mm. (**b**) The thickness of the flexible plate is set to 0.7 mm. (**c**) The flexible plate’s thickness is 0.9 mm.

In this part of the experiment, the flapping frequency of the foil is set to 1.0 Hz, and the rest of the experimental parameters are kept the same as in the above experiments. In Figure 5, the experimental results show the propulsive force changes in the range of 0 to 8 N. Besides, the thicknesses of the flexible plate used in Figure 5a–c are 0.5 mm, 0.7 mm, and 0.9 mm, respectively. As can be seen from Figure 5a, the 3-1 foil produces the largest propulsive force, while the low stiffness (0-4) foil produces the relatively smallest thrust. In Figure 5b, the 2-2 foil has the relatively largest propulsive force, and the same 0-4 foil has the smallest propulsive force. The information in Figure 5c shows that the relatively largest propulsive force produces by the 2-2 foil, while the 0-4 foil has the smallest thrust.

**Figure 6:**
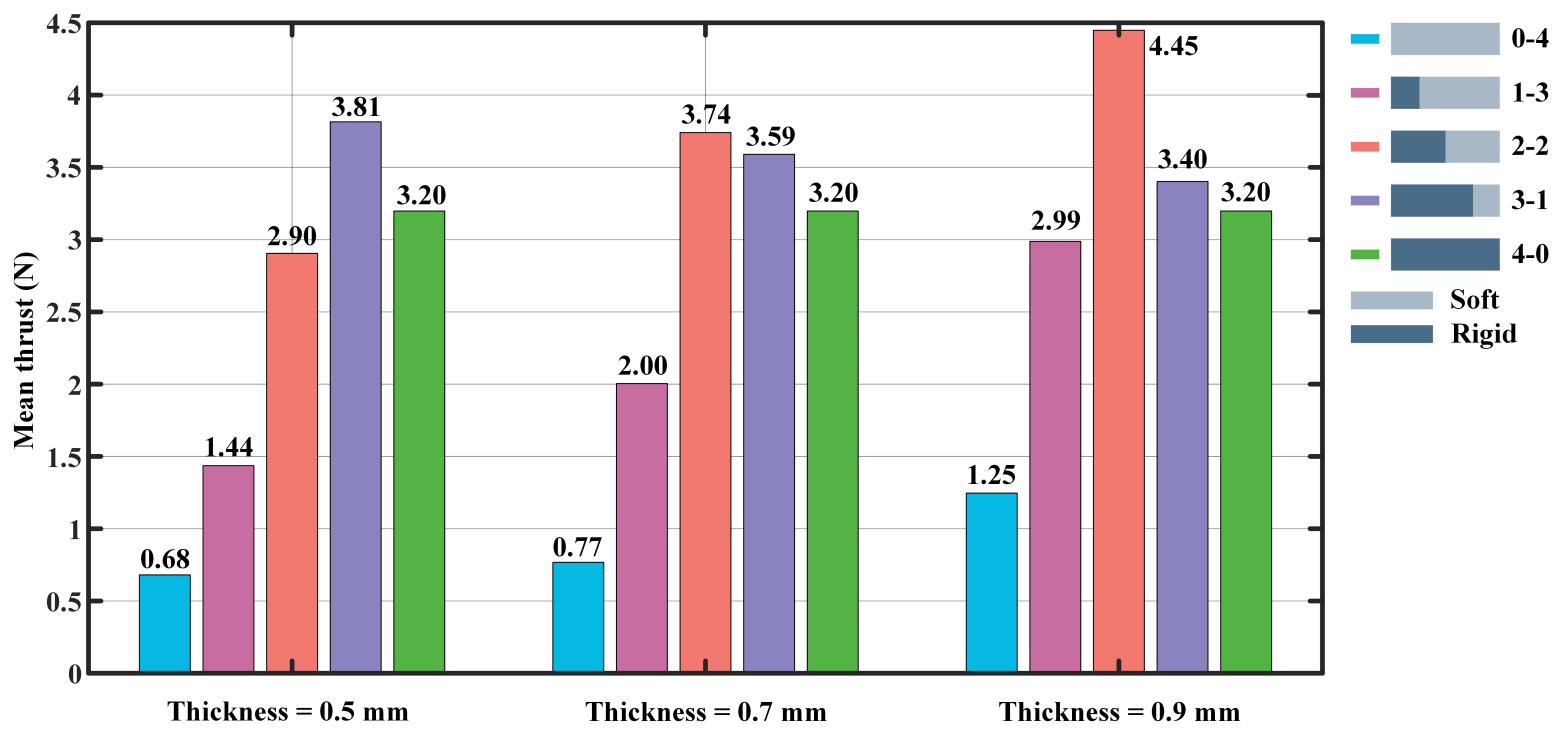
The mean thrust produced by the foil at the flapping frequency of 1 Hz, averaged over all flapping angles from 20° to 50°.

From Figure 6, there is an image of the mean thrust for the flapping frequency set to 1.0 Hz, and the flapping angles of the foil are set at 20°, 30°, 40°, and 50°, respectively. Similarly, the thicknesses of flexible plates used in the experiments are 0.5 mm, 0.7 mm, and 0.9 mm. The experimental results show that the largest average thrust is provided by the 3-1 foil with a value of 3.81 N, while the smallest propulsive force is provided by the 0-4 foil with a value of 0.68 N when the thickness of the flexible plate is 0.5 mm. In addition, when the flexible plate’s thickness is set to 0.7 mm, the largest average propulsive force is 3.74 N and the smallest value is 0.77 N, generated by the 2-2 and low stiffness (0-4) foils, respectively. When the thickness is 0.9 mm, the largest average thrust force of 4.45 N is provided by the 2-2 foil, while the smallest thrust of 1.25 N is produced by the low stiffness (0-4) foil. Overall, the propulsive force generated by the 0-4, 1-3, and 2-2 foils gradually increase with the increase of the flexible plate’s thickness. In contrast, there is a significant decline in the propulsive force generated by the 3-1 foil. Furthermore, the 2-2 foil produces the largest mean thrust of 4.45 N, while the propulsive forces of the low stiffness (0-4) and high stiffness (4-0) foils are 1.25 N and 3.20 N, respectively. It can be found that the rigid-flexible model foils produce a larger propulsive force, compared to the purely flexible or rigid foils. If the thickness of the flexible plate increase, the thrust will increase.

### 3.3. Experiment III (Frequency = 1.5 Hz)

**Figure 7:**
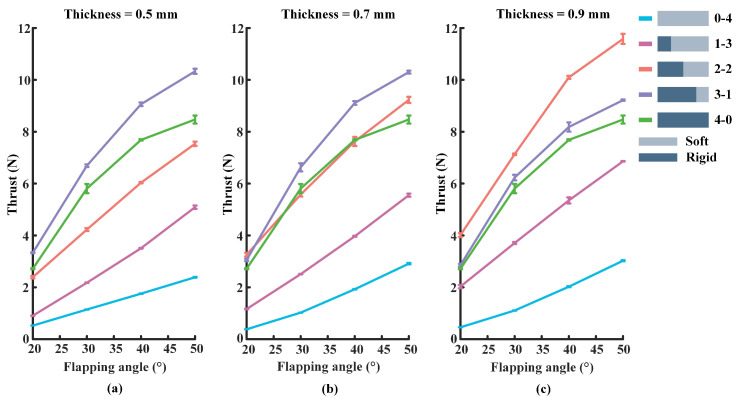
The thrust produced by the foil at the flapping frequency of 1.5 Hz: (**a**) The thickness of the flexible plate of the foil is set to 0.5 mm. (**b**) The thickness of the flexible plate is set to 0.7 mm. (**c**) The flexible plate’s thickness is 0.9 mm.

The flapping frequency of the foil is set to 1.5 Hz in this experiment, and the rest of the experimental parameters are kept consistent with Section 3.1 and Section 3.2. Figure 7 shows the propulsive force generated by the different foils. Overall, the value of the propulsive force varies in the range of 0 to 12 N. As can be seen from Figure 7a, When the thickness of the flexible plate is 0.5 mm, the 3-1 foil produces the largest propulsive force, while the low stiffness foil (0-4) has the smallest thrust. Furthermore, the information in Figure 7b reveals that the largest propulsive force is generated by the 3-1 foil, and the low stiffness (0-4) foil produces the smallest thrust when the thickness of the flexible plate is set to 0.7 mm. From Figure 7c, when the flexible plate’s thickness is 0.9 mm, the 2-2 foil produces the largest propulsive force, and the propulsive force produced by the 0-4 foil is still the smallest.

**Figure 8:**
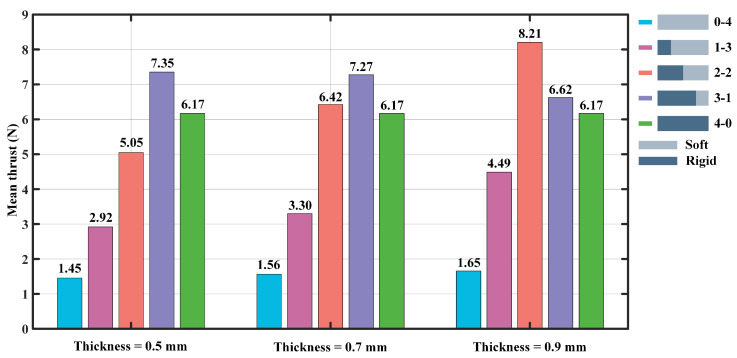
The mean thrust produced by the foil at the flapping frequency of 1.5 Hz, averaged over all flapping angles from 20° to 50°.

Figure 8 shows the mean thrust information for the flapping angle of 20° to 50°. It can be found that when the thickness of the flexible plate is 0.5 mm, the largest mean propulsive force is 7.35 N, reached at the 3-1 foil, and the smallest mean propulsive force is 1.45 N reached at the 0-4 foil. When the thickness of the flexible plate is 0.7 mm, the largest mean propulsive force is 7.27 N, reached at the 3-1 foil, and the smallest mean propulsive force is 1.56 N, reached at the 0-4 foil. When the thickness of the flexible plate is 0.9 mm, the largest mean propulsive force is 8.21 N, reached at the 2-2 foil, and the smallest mean propulsive force is 1.65 N, reached at the 0-4 foil. Overall, the propulsive force gradually increases generated by the 0-4, 1-3, and 2-2 foils as the increase of the flexible plate’s thickness. In contrast, there is a significant decline in the propulsive force generated by the 3-1 foil. In addition, when the flexible plate’s thickness is 0.9 mm, the 2-2 foil produces the largest mean propulsive force of 8.21 N, while the low stiffness (0-4) and high stiffness (4-0) foils provide mean propulsive forces of 1.65 N and 6.17 N, respectively.

**Figure 9:**
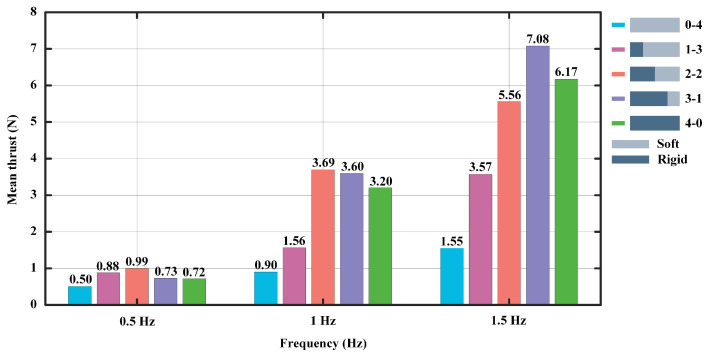
The mean thrust of each model.

To demonstrate the experimental results more clearly, we take the mean values of the experimental results for each model in Figure 4, Figure 6 and Figure 8, which will no longer show the effect of the thickness of the flexible foil on the propulsive force. As shown in Figure 9, when the motion frequency is set to 0.5 Hz, the 2-2 foil produces a maximum average propulsive force of 0.99 N. When the frequency is 1 Hz, the result is consistent with the previous one, with an average propulsive force of 3.69 N. When the frequency is set to 1.5 Hz, the 3-1 foil produces the maximum average propulsive force of 7.08 N. In general, it is clear that the longer flexible posterior border provides more propulsion force at a low frequency, while the longer rigid anterior border performs better at a high frequency.

## 4. Discussion

The main reason for the poor propulsion performance of the traditional “rigid” biomimetic robotic fish is that the body cannot yet adjust its stiffness like a real fish. In nature, the stiffness of a real fish decreases along its axial direction from the head to the tip of the caudal fin, and its non-unitary stiffness plays a crucial role in the modulatory of propulsive force. Numerous experiments by previous researchers have proven that if the body of the biomimetic robotic fish uses pure rigid material, the propulsion performance is partially improved, but not significantly. Excessive rigidity of the material will severely limit the movement flexibility of the robotic fish. In addition, although the purely flexible material is helpful to enhance the flexibility of movement, its propulsion performance will not be able to meet the working requirements when the robotic fish is under high-frequency movement. Currently, a large number of experimental studies on uniform and non-uniform stiffness have been conducted, but the optimal stiffness required to maximize propulsive force has not been found. In this paper, the research idea of variable non-uniform stiffness material is applied to investigate the method of improving the propulsive force.

Experimental results show that a lower non-uniform stiffness ratio will provide a higher propulsive force of motion when the robotic fish is at a low-frequency swimming speed. Specifically, the non-uniform stiffness material labeled 1-3 (flexible plate’s thickness is 0.9 mm) produces the largest average propulsive force with a value of 1.11 N when the flapping frequency of the foil is set to 0.5 Hz. Moreover, as the thickness of the flexible material increases, the propulsive force will also become larger. When in a high-frequency motion, the foil needs a high non-uniform stiffness ratio to achieve the largest propulsive force. Specifically, the 2-2 foil (the flexible plate’s thickness is set at 0.9 mm) produced the largest average propulsive force of 8.21 N when the foil flapping frequency is 1.5 Hz. Why is there such a big difference in the propulsive force generated by different non-uniform stiffness models when switching between low and high frequencies?

**Figure 10:**
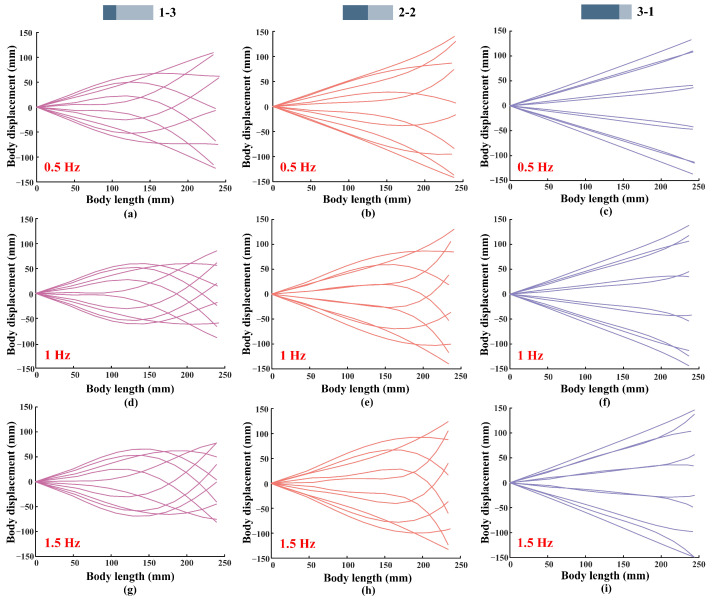
The displacement curves of the models. The flapping frequencies of the foil are set to 0.5 Hz, 1 Hz, and 1.5 Hz, the flapping amplitude is fixed at 30°, and the thickness of the flexible posterior border is 0.5 mm.

**Figure 11:**
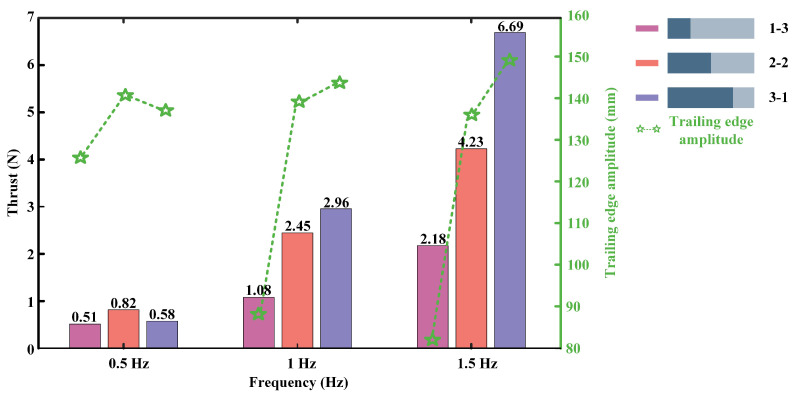
Comparison of thrust and trailing edge amplitude variation trends.

As shown in Figure 10, we obtained the displacement curves of the models. The flapping frequencies of the foil are set to 0.5 Hz, 1 Hz, and 1.5 Hz, the flapping amplitude is fixed at 30°, and the thickness of the flexible posterior border is 0.5 mm. As shown in the figure, when the foil flapping frequency is set to 0.5 Hz, the 2-2 foil has the largest amplitude of the trailing edge, followed by the 3-1 foil, and the smallest oscillating amplitude is produced by the 1-3 foil. When the flapping frequency is 1 Hz, the 3-1 foil has the largest trailing edge amplitude, followed by the 2-2 foil, and the 1-3 foil produces the smallest trailing edge amplitude. When the frequency is set to 1.5 Hz, the results remain the same as when the frequency is 1 Hz. To further obtain the effect of trailing edge amplitude on the propulsive force, we make a comparison graph of the trend of thrust and trailing edge amplitude, as shown in Figure 11. We surprisingly found that the variation of thrust and trailing edge amplitude are positively correlated regardless of the variation of motion frequency. In addition, comparing the envelopes of the same model with different motion frequencies, as shown in Figure 10a,d,g, it can be found that when the motion frequency increases, the trailing edge amplitude of 1-3 foil will decrease. While comparing Figure 10c,f,i, it can be found that increasing the frequency causes an increase in the trailing edge amplitude. This explains why the longer flexible posterior border provides more propulsion force at a low frequency, while the longer rigid anterior border performs better at a high frequency. In addition, the experimental results are consistent with the views discussed in Lucas’s paper [10], but only when the proportion of rigid plate in the non-uniform foil is larger.

It is worth noting that no matter how the thickness of the flexible material of the foil varies, the propulsive force generated by the 3-1 (stiff anterior three quarters) foil will be at a high level, and its propulsive force output is more stable at the same motion frequency. The emergence of this experimental result may be related to the active and passive flexural stiffness of the creatures doing propulsive motions. The literature [10,33] explored the flexural stiffness state of a variety of creatures during propulsive motion. The actuators of most organisms were found to be bent at 2/3, which gives a reasonable explanation for the current experimental results we obtained. From the analysis of the above results, it is clear that the stiffness needs to be adjusted accordingly with the change of the motion parameters, and a fixed stiffness does not contribute much to the propulsive force.

Currently, many efforts have been made to find the optimal stiffness used to drive the biomimetic robotic fish, but neither uniform stiffness nor non-uniform stiffness nor variable stiffness studies with “artificial muscles” have found the optimal stiffness. What has been demonstrated in the concept of variable non-uniform stiffness of the foil material presented in this paper is that a different movement frequency requires adjustments to the non-uniform coupling ratio of the foil to achieve the relative maximization of the propulsive force of the robotic fish. In the future, biomimetic robotic fish’s material stiffness should be researched along the direction of “dynamic” and “non-uniform stiffness”, which has highly bionic significance.

## 5. Conclusions and Future Work

In this paper, we conduct an experimental study on how non-uniform stiffness affects the propulsion performance of a biomimetic robotic fish. A customized experimental platform, which eases the adjustment of the non-uniform stiffness ratio, the stiffness of the flexible part, the flapping frequency, and the flapping amplitude, is built, and extensive experiments are carried out. It is found that to maximize the propulsion performance of the biomimetic robotic fish, the non-uniform stiffness ratio needs to be reduced when the robotic fish works at low frequency, and it needs to be increased when the robotic fish works at high frequency. Specifically, when the flapping frequency is 0.5 Hz, the largest average propulsive force of 1.11 N is generated by the foil with a 1-3 non-uniform stiffness ratio. When the flapping frequency is 1.0 Hz, the largest average propulsive force of 4.45 N is generated by the foil with a 2-2 non-uniform stiffness ratio. When the flapping frequency is 1.5 Hz, the largest average propulsive force of 8.21 N is generated by the foil with a 2-2 non-uniform stiffness ratio.

In the future, more factors, such as the shape, the size, and the flapping pattern, will be taken into consideration. Moreover, the stiffness adjustment mechanism to change the non-uniform coupling ratio online, and its influence on propulsion efficiency, speed, and maneuverability, will also be investigated. 

## Data Availability

The datasets generated during and/or analyzed during the current study are available from the corresponding author on reasonable request.
